# Air Below the Right Diaphragm

**DOI:** 10.4103/1319-3767.56088

**Published:** 2009-10

**Authors:** Aaisha Khan, Sarmad Waqas

**Affiliations:** Post Graduate Trainee, Mayo Hospital, Lahore, Pakistan

## CASE SCENARIO

**Figure 1:**
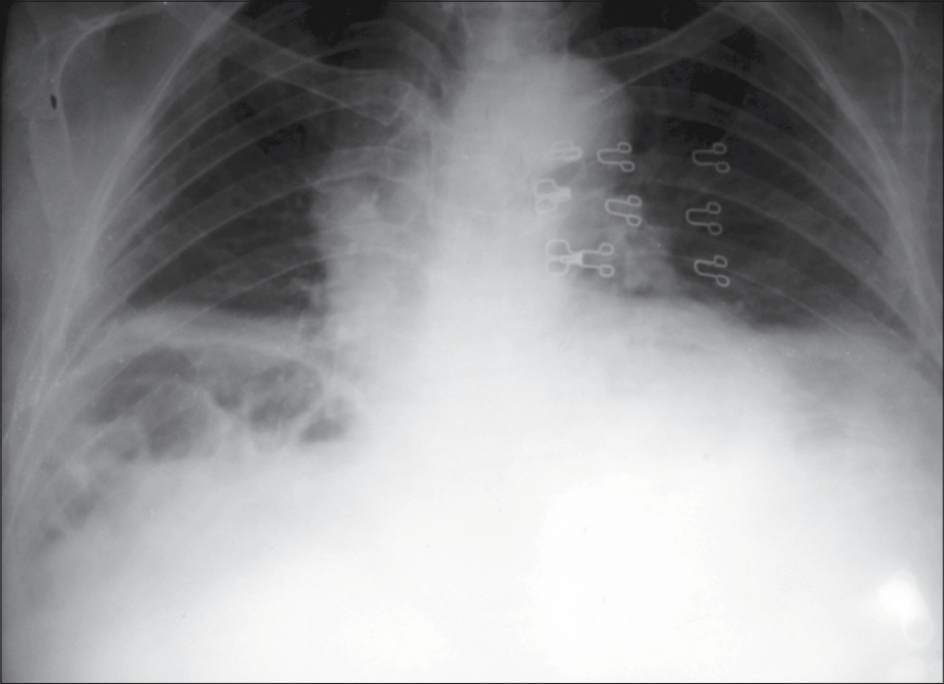
X-ray

A young woman aged 25 years presented to the emergency department with nausea and abdominal pain for a couple of hours. She had no medical or surgical history. On examination, her vitals were normal. There was some tenderness in the epigastric region, but other than that no significant clinical findings were noted. Electrocardiogram and all the routine blood tests were normal. Her chest X-ray (posteroanterior view) was done and is shown below [Figure 1]. She had no previous X-rays.

## QUESTIONS

1. Describe the radiological sign shown in the Figure 1?
2. What is the difference between the associated sign and the syndrome?
3. Name a few conditions associated with this sign?
4. How will this patient be managed?

## ANSWERS

1. This chest X-ray (anteroposterior view) shows interposition of the small bowel between the liver and the right hemidiaphragm, and radiologically this term is called Chilaiditi's sign.[1] It was first described by Demetrious Chilaiditi in 1910,[2] who discovered 3 cases of hepatodiaphragmatic interposition (HDI). Since then it is known as Chilaiditi's sign.[1] Chilaiditi's sign is generally an incidental, asymptomatic radiographic finding.[1] It may remain undiagnosed throughout ones lifetime.[3] Chilaiditi's sign is recognized on plain X-ray chest by air collection marking with haustral signs in the subdiphragmatic area. If in doubt, CT chest with contrast can differentiate a subdiaphragmatic abscess from it.[3]
2. Chilaiditi's sign is an x-ray finding of hepatodiaphragmatic interposition. However, in some people, it is associated with symptoms such as nausea, abdominal pain, vomiting, distention, flatulence, substernal pain, incomplete intestinal obstruction, cardiac arrhythmias or dyspnea.[3] If symptomatic, this radiographic term is called Chilaiditi's sign.[2]
3. Chilaiditi's sign is noted in many disorders including liver cirrhosis, chronic obstructive lung disease, near-term pregnancy, mental disorders and obesity. Chilaiditi's syndrome has been reported with colonic volvulus, suprahepatic appendicitis, scleroderma, congenital hypothyroidism, melanosis coli and salmonellosis. A few articles mention Chilaiditi's syndrome with breast, colonic, gastric and lung carcinoma.[3]
4. Mostly symptoms resolve by bed rest and conservative management. If symptoms progress, patient should be referred to surgery.[3]
